# How to choose the therapeutic goals to improve tissue perfusion in septic shock

**DOI:** 10.1590/S1679-45082015RW3148

**Published:** 2015

**Authors:** Murillo Santucci Cesar de Assuncao, Thiago Domingos Corrêa, Bruno de Arruda Bravim, Eliézer Silva

**Affiliations:** 1 Hospital Israelita Albert Einstein, São Paulo, SP, Brazil.

**Keywords:** Shock, septic, Hemodynamics, Resuscitation, Sepsis, Vasoconstrictor agents, Microcirculation

## Abstract

The early recognition and treatment of severe sepsis and septic shock is the key to a successful outcome. The longer the delay in starting treatment, the worse the prognosis due to persistent tissue hypoperfusion and consequent development and worsening of organ dysfunction. One of the main mechanisms responsible for the development of cellular dysfunction is tissue hypoxia. The adjustments necessary for adequate tissue blood flow and therefore of oxygen supply to metabolic demand according to the assessment of the cardiac index and oxygen extraction rate should be performed during resuscitation period, especially in high complexity patients. New technologies, easily handled at the bedside, and new studies that directly assess the impact of macro-hemodynamic parameter optimization on microcirculation and in the clinical outcome of septic patients, are needed.

## INTRODUCTION

Sepsis is characterized by the heterogeneous pattern of blood flow in microcirculation, by tissue hypoperfusion and by incapacity of the cells to extract and adequately use oxygen, which compromises the aerobic cell metabolism.^(1-3)^ Imbalance between oxygen delivery (DO_2_) and the metabolic cell demand is what characterizes the state of shock.^(4)^

Sepsis, associated with at least on organ dysfunction, is called severe sepsis. Septic shock is defined by the need for a vasopressor to maintain perfusion pressure after adequate fluids resuscitation.^(5)^

Sepsis compromises the cardiovascular system by various mechanisms that frequently result in alterations of tissue perfusion. Tissue hypoperfusion contributes towards the development of cell dysfunction, which can progress to multiple organ failure and death.^(6)^ The primary objective of treatment in states of shock is reestablishment of DO_2 _to the tissues, in order to guarantee adequate tissue oxygenation demand. Thus, early and rapid interventions designed to revert tissue hypoperfusion were associated with reduced mortality in septic patients.^(5)^

The objective of this review of literature is to discuss the main hemodynamic and laboratory parameters that direct the bedside approach of the patient with septic shock.

## CLINICAL AND MACROHEMODYNAMIC PARAMETERS

It is known that clinical and macrohemodynamic parameters show a low correlation with the state of tissue perfusion. In 1996, Rady et al. demonstrated the presence of occult tissue hypoperfusion by the presence of hyperlactatemia or reduction in central venous oxygen saturation (ScvO_2_).^(3)^ Critically ill patients were submitted to initial resuscitation with fluids, followed by stabilization of clinical parameters. Approximately 86% of patients evaluated presented with hyperlactatemia or SvcO_2_<65% after stabilization of the clinical parameters.^(3)^ This was characterized as occult tissue hypoperfusion or cryptic shock.^(7)^

Such findings make it clear that clinical parameters should not be used as a therapeutic goal in resuscitating critically ill patients. The study done by Rady et al.^(3)^ preceded the Early Goal-Directed Therapy, which demonstrated that a group of interventions promptly performed involving infusion of fluids, vasopressors, inotropics, and red blood cell transfusion, with the goal of achieving SvcO_2_≥70%, reduced mortality in patients with severe sepsis and septic shock.^(8)^

The results of this study are considered the greatest evidence available that optimization of tissue perfusion should be performed quickly and should be added as a goal in the treatment of this population of severely ill patients.^(8) ^However, such a benefit was not observed in two large studies published recently.^(9,10)^ The negative results obtained in these two studies may be, at least partially, justified by the fact that the patients included soon received an infusion of fluids, before randomization.^(9,10)^ The study by Rivers et al. was the major turning point in the approach of critically ill patients having as primary message the importance of early intervention in this population.^(8)^ After this study and with the dissemination of information as to the need for early recognition and treatment of patients with severe sepsis or septic shock, it becomes difficult to compare a management approach that changed the therapeutic approach. Additionally, delay in recognizing and treating this population of severely ill patients is considered unethical today.

Both the ProCESS study as well as the ARISE (Australasian Resuscitation in Sepsis Evaluation) had as inclusion criteria the need for infusion of fluids and initiation of wide spectrum antibiotics.^(9,10) ^These measures emphasized the importance of the timeliness of treatment and certainly contributed towards the observation of similar mortality among the several groups studied.^(9,10)^ Early initiation of treatment is what differentiates these two investigations from the study conducted by Rivers et al.^(8-10)^

Various parameters and markers of blood flow and systemic tissue perfusion are available at bedside, as well as mixed venous oxygen saturation (SvO_2_), lactate clearance, DO_2_, and oxygen consumption (VO_2_). The interpretation of these markers is necessary for adequate resuscitation and reestablishment of tissue perfusion.

## OXYGENATION PARAMETERS

SvO_2_ reflects the total oxygenated blood that returns to the right heart via right atrial drainage, through the superior vena cava, inferior vena cava, coronary sinus, and the veins of Thebesius.^(11)^ SvO_2_ is measured in a sample of blood collected from the pulmonary artery. The venous blood from various parts of the body starts to homogenize in the right atrium and as it progresses towards the lungs, it becomes increasingly more homogeneous. Once it reaches the pulmonary artery, the venous blood from all parts of the body is totally “mixed”, that is, completely homogenized, and thus receives the name “mixed venous blood.”^(11)^ SvcO_2_, on the other hand, corresponds to oxygen saturation by the blood hemoglobin that is found at the blood drainage from the superior vena cava into to the atrium. SvcO_2 _reflects the quantity of oxygen that returns to the right heart from the upper limbs, neck, and head.^(11)^

In healthy individuals, SvcO_2_ has a mean value of 77%, with a variation between 66 and 84%, while SvO_2_ has a mean value of 78%, with variation between 73 and 85%. Thus, in normal situations, SvcO_2_ has lower values relative to those of SvO_2_.^(11)^ Nevertheless, due to greater VO_2 _in the splanchnic circulation, in intra-abdominal organs and lower limbs in states of shock, SvcO_2_ values exceed those of SvO_2_.^(11)^

**Figure 1:**
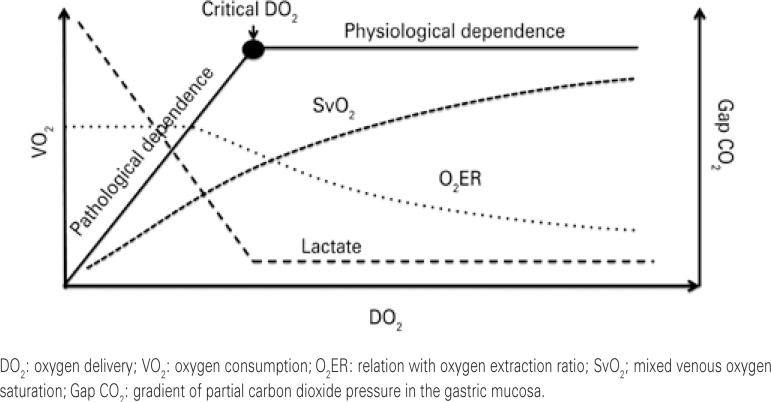
Relations between supply, consumption, and oxygen extraction ratio, mixed venous saturation of oxygen, arterial lactate, and gradient of partial carbon dioxide pressure in the gastric mucosa

Considering that arterial oxygen saturation is constant, SvO_2_ shows a direct relation with cardiac output (Figure 1). In addition to cardiac output (systemic flow), SvO_2_ has an inverse relation with oxygen extraction ratio (O_2_ER) (Figure 1). On the other hand, O_2_ER means the quantity of oxygen that the cells can extract from a given quantity of oxygen supplied and it is the ratio between VO_2_ and DO_2_.^(4)^

DO_2_ is related to blood flow and the quantity of oxygen bound to hemoglobin and dissolved in plasma, and is defined as the quantity of oxygen that reaches the cells to supply the body metabolic demand. The quantity of oxygen that the cells consume is related to the capacity of cell extraction of oxygen. In this way, VO_2_ is defined by the quantity of oxygen used by the cells for the production of energy in order to supply the metabolic demand.^(4)^

Physiologically, every time there is a reduction in DO_2_, there will be an increase in tissue oxygen extraction in order to maintain the VO_2_ stable (Figure 1). In this situation, it is expected that SvO_2_ decreases, since it means the total oxygenated blood that returns from the systemic circulation to the right heart and reflects the balance between VO_2_ and DO_2_. However, when reductions in DO_2_ are accompanied by decreased VO_2_, the mechanism of anaerobiosis is initiated to supply the metabolic demand of the body. Within this scenario, the increased oxygen extraction is not capable of supplying or maintaining the oxygen-dependent metabolic needs. When this process begins, DO_2_ is called critical DO_2_ and VO_2_/DO_2_ dependence is established (Figure 1).^(4)^

Despite VO_2_/DO_2_ dependence not being frequently associated with states of high-level metabolism, such as in sepsis, it may be present at the end of resuscitation guided by ScvO_2_.^(4,12)^ Optimization of systemic blood flow guided by ScvO_2_ during the initial six hours of resuscitation of patients with severe sepsis or septic shock reduced mortality of this population of patients when compared to the control group.^(8)^ However, ScvO_2_ represents a systemic adjustment marker of blood flow, and values of ScvO_2_≥70% do not mean that the regional blood flow, in each organ and tissue, is not completely optimized.^(8)^ To be sure that the initial resuscitation reached its goal, one should exclude the presence of oxygen deficit, which is characterized by increased VO_2_ due to optimization related to increase of blood flow.^(4)^

What should always be questioned during resuscitation is if the blood flow obtained is compatible with the metabolic demand for oxygen. For this, a test has been suggested as a method for assessing the existence of oxygen debit, in which the cardiac output is increased and consequently, tissue oxygen flow as well. The absence of SvO_2_ alteration after increasing the cardiac output suggests an increase in VO_2,_ meaning that there is yet a oxygen debit and therefore, that the resuscitation has not yet reached the primary objective.^(13-17)^

To reach the adjustment of the DO_2_/VO_2_ ratio is the rationale for the correction of hypoperfusion. Evaluation of the relation between O_2_ER and the cardiac index allows optimization of VO_2_.^(4)^ With the objective of adequately adjusting DO_2_ to VO_2_, high values of DO_2 _may be needed. This high value of DO_2 _for a high value of VO_2_ is not synonymous of supranormal therapy. In supranormal therapy, preestablished high values of DO_2_ are the goal, regardless of the DO_2_/VO_2 _ratio.^(14-16)^

**Figure 2:**
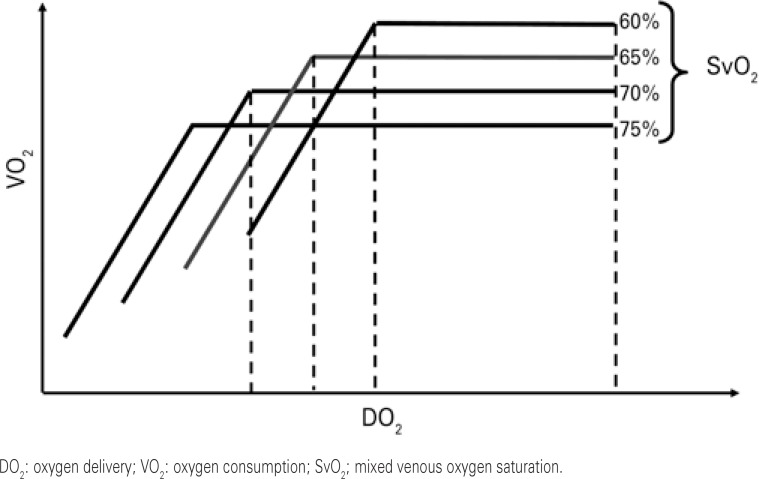
Behavior of mixed venous saturation of oxygen, according to supply and consumption of oxygen

During the acute phase of septic shock, DO_2_/VO_2_ dependence may be identified and corrected.^(12)^ An adequate DO_2_/VO_2 _ratio, is therefore, the most important endpoint of resuscitation. In adding cardiac output increments that lead to increased VO_2_, one can infer that areas that were not being adequately perfused started to be perfused and there was an increase of VO_2_, that is, a recruitment of microcirculation (Figure 2). Adjustment of the cardiac index to the metabolic demand, associated with normalization of the systemic perfusion parameters, such as SvO_2_ or lactate, allow the deduction that the resuscitation was concluded successfully.

Based on the three pillars that have greatest impact on cardiac output, that is, preload, contractility, and afterload, optimization of blood flow can be initiated by evaluating the fluid responsiveness. The benefit of fluid infusion occurs when the patient is on the ascending portion of the Frank-Starling curve, that is, the patient still presents with recruitable preload. If the patient is on the Frank-Starling curve plateau, *i.e*., does not respond to fluid of infusion, it is possible to use inotropic agents, such as dobutamine, with an initial dose of 2.5mcg/kg/min, evaluating gain of flow by the increase of the cardiac index, always associated with analysis of O_2_ER in order to infer the behavior of VO_2_. In this way, regardless of the strategy adopted, the increased blood flow with the maintenance of SvO_2 _values, translated by the increase in O_2_ER would imply an increase of VO_2_ (Figures 1 and 2).

Integrated analysis of various hemodynamic parameters seems to be the most adequate and useful in the treatment of patients in shock, in comparison with the isolated use of one of the variables.

## LACTATE CLEARANCE

Lactate is a marker of systemic tissue perfusion and is elevated in cases of hypoperfusion of the tissues. Delay in normalizing lactate in severely ill patients is an indicator of unfavorable prognosis. Within this context, the duration of the hyperlactatemia is more important than the baseline value of the lactate.^(18)^ However, in sepsis, there are other factors in addition to systemic hypoperfusion that can contribute towards hyperlactatemia, such as dysfunction of the pyruvate dehydrogenase enzyme, mitochondrial dysfunction, and the presence of hepatic or renal dysfunction.^(19)^

Lactate clearance is represented by the percentage of reduction in levels of lactate soon after the start of resuscitation. Lactate clearance ≥10% was associated with better prognosis in severely ill patients, with reduced mortality among patients with severe sepsis and septic shock.^(20)^

**Figure 3:**
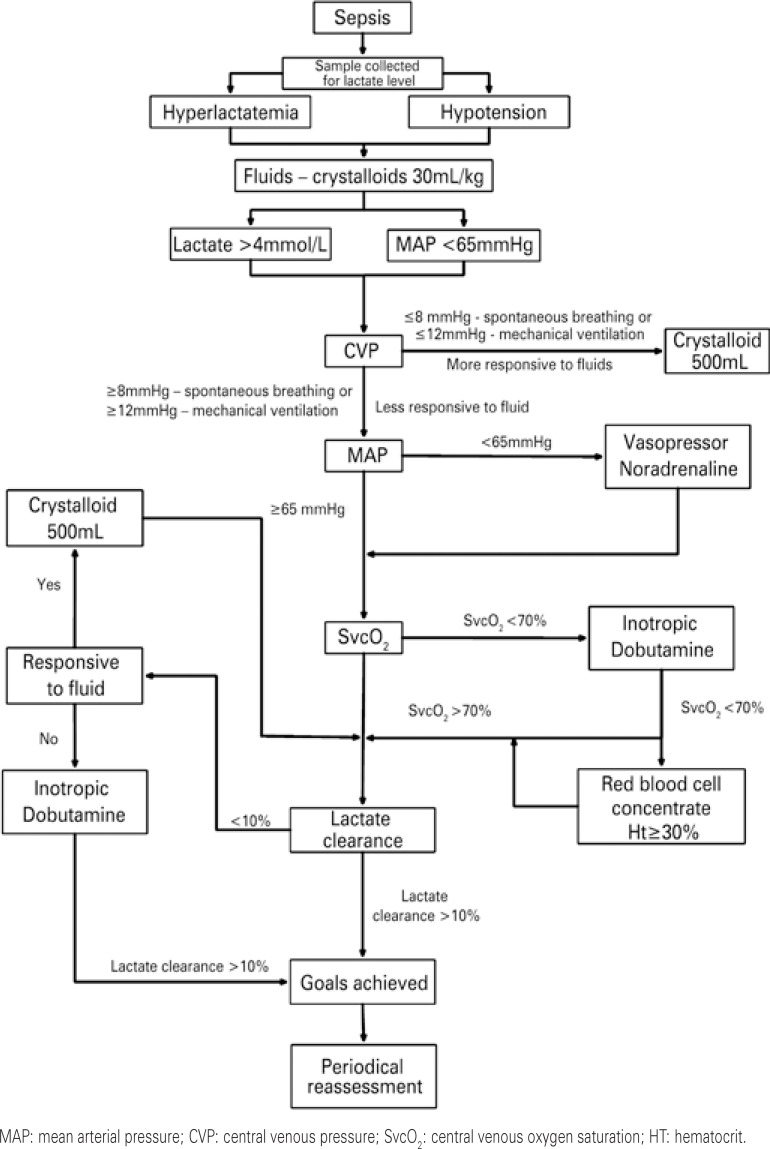
Goal-guided resuscitation algorithm protocol for patients with septic shock

Lactate clearance is a parameter to be monitored during optimization of systemic tissue perfusion.^(20-22)^ The guidelines of the Surviving Sepsis Campaign recommend accompaniment of lactate clearance and its normalization as early as possible, during the initial phase of resuscitation (Figure 3).^(5)^

Lactate clearance may be used as a surrogate SvcO_2_ monitoring, for optimization of the systemic blood flow during the initial phase of resuscitation of patients with severe sepsis and septic shock.^(23)^ The use of an aggressive resuscitation protocol, with the objective of clearing the lactate by at least 20%, every 2 hours, significantly reduced length of stay at hospital, in the intensive care unit (ICU), and hospital mortality.^(24)^ Nevertheless, there is no evidence yet that normalization of lactate presents with additional benefits when compared to resuscitation guided only by central venous pressure (CVP), mean arterial pressure (MAP), diuresis and SvcO_2_.

However, normalization of lactate levels can not be defined as an endpoint for microcirculation resuscitation. The lactate measured corresponds to the dilution of global production of lactate by all organs. This means that there may be regional tissue hypoperfusion, even when the plasma levels of lactate are normalized.

## MICROHEMODYNAMIC PARAMETERS

### Regional blood flow

In sepsis, the variables of regional perfusion are altered early relative to the systemic variables, and after resuscitation, the regional variables can be the last ones to normalize.^(25,26)^ These variables are the gastric mucosa pH and the gradient between the partial pressure of carbon dioxide of the gastric mucosa (Gap CO_2_) and the partial pressure of carbon dioxide (PCO_2_ gap).

Gastric tonometry or sublingual capnometry can be used for early detection of tissue hypoperfusion. An increase in the CO_2_ gap may reflect abnormal blood flow in the gastric mucosa. In this way, monitoring by gastric tonometry could help in the identification and correction of regional circulatory abnormalities.

However, no study has been able to confirm the benefits of the use of these techniques as a therapeutic guide, especially in septic patients, and currently, gastric tonometry is a technique used only in experimental models of shock.^(27)^

### Microcirculation dysfunction: a new field to be explored

Under normal conditions, only 30% of the capillaries are perfused. In this way, it is possible to imagine that, even in normal conditions, microcirculation does not present with homogeneous oxygenations. In order to achieve a homogenous oxygenation, the tissues need to recruit capillaries, which normally occurs in conditions of metabolic stress.^(28)^

Microcirculation dysfunction in severe sepsis and in septic shock is characterized by the presence of abnormalities in blood flow, and areas with high blood flow and normal tissue perfusion can coexist with areas with altered perfusion. The presence of hypoperfused capillaries can compromise tissue oxygenation and contribute towards the development of multiple organ failure. It is currently considered that the reversal of these microcirculatory disturbances can be a therapeutic goal to be sought during resuscitation from sepsis, which would cause the reversal of the areas of hypoxia resulting from tissue hypoperfusion triggered by the great number of shunts. Thus, microcirculation dysfunction has become a concern in treatment of septic patients and its reversal has become a therapeutic goal in resuscitation from septic shock.

Two hypotheses were also raised as to the origin of the microcirculation dysfunction in septic shock. First, alteration and loss of self-regulation of microcirculation would result in heterogeneous perfusion, which would promote areas of hypoxia, resulting in an increase in shunt areas.^(29)^ Second, mitochondrial dysfunction could be induced by sepsis, which could alter the mitochondrial capacity to use oxygen in spite of adequate tissue oxygenation (cytopathic hypoxia).^(30)^ Nonetheless, the extensive endothelial lesion, release of inflammatory mediators, activation of coagulation, and alterations in cell metabolism of oxygen can contribute towards the development of microcirculation dysfunction in patients with septic shock.^(31)^

The degree of microcirculation dysfunction is an independent predictor of survival. Septic patients with severe alterations in sublingual microcirculation present with the worst prognosis relative to those who do not show significant alterations in microcirculation.^(1,32)^ In an observational study conducted during the first six hours of resuscitation of patients with severe sepsis and septic shock, the compromise of the microcirculation perfusion indexes was associated with reduced survival.^(33)^ In this study, improved blood flow in the microcirculation was associated with a lower incidence of organic dysfunction in 24 hours, with no significant alterations in systemic hemodynamic parameters detected.^(34)^ Hence recruitment of microcirculation aiming to improve cell oxygenation has the potential to minimize the progression of organic dysfunction, by satisfying and adjusting cell oxygenation according to the metabolic demand.

Some vasoactive drugs can optimize microcirculation perfusion. Dobutamine improves capillary perfusion, regardless of the macro-hemodynamic parameters. The reduction in lactate levels correlates with improved microcirculation, regardless of cardiac index.^(35)^ However, lactate is not a marker of microcirculation resuscitation.

Another way to promote recruitment of capillaries is the use of vasodilation agents. In sepsis, it was proposed that the use of vasodilators may increase blood flow in microcirculation, increasing perfusion in areas of hypoxia.^(36)^ Nonetheless, there are still controversies as to the use of vasodilators for recruitment of microcirculation. Vasodilators (nitroglycerin and sodium nitroprusside) are nitric oxide donors’ agents, which could contribute to cell dysfunction of sepsis. While Spronk et al. demonstrated improved perfusion of sublingual microcirculation with infusion of nitroglycerin,^(37)^ Boerma et al. reported that the use of nitroglycerin did not improve the sublingual microcirculation perfusion in comparison with the administration of placebo.^(38)^ However, there was a tendency towards an increase in hospital mortality in the group of patients that received nitroglycerin, even though this study did not have power enough to confirm the association between the administration of nitroglycerin and increased mortality. Another interesting finding of this study was that the patients on nitroglycerin presented with a lower number of organic dysfunctions when compared to the placebo group.^(38)^

Resuscitation and recruitment of microcirculation with administration of fluids, dobutamine and vasodilators may be a promising strategy. However, the assessment of microcirculation at the bedside continues limited to research protocols, despite the fact that, over the last years, new equipment for evaluation of microcirculation has been developed, facilitating the use of this bedside technology.^(39)^ In addition to improvement of the techniques available, there is yet the need for adequate clinical trials to confirm the true benefit of this resuscitation strategy.

## CONCLUSION

Early optimization of tissue oxygenation is mandatory in resuscitation of patients with severe sepsis and septic shock. The use of an early resuscitation protocol guided by goals, which includes central venous oxygen saturation or lactate clearance as parameters of adequate blood flow and consequently, of supply of oxygen to the metabolic demand, is highly recommended by the guidelines that direct the resuscitation of patients with severe cases of sepsis and septic shock.

New technologies for direct visualization of the microcirculation, easy to use at the bedside and cost-effective, are still needed for greater use in daily clinical practice. Additionally, new studies, with the objective of evaluating the impact of optimization of macrohemodynamic parameters on microcirculation and on the outcome of septic patients are still necessary.

## References

[1] Backer D, Creteur J, Preiser JC, Dubois MJ, Vincent JL (2002). Microvascular blood flow is altered in patients with sepsis. Am J Respir Crit Care Med.

[2] Jeger V, Djafarzadeh S, Jakob SM, Takala J (2013). Mitochondrial function in sepsis. Eur J Clin Invest.

[3] Rady MY, Rivers EP, Nowak RM (1996). Resuscitation of the critically ill in the ED: responses of blood pressure, heart rate, shock index, central venous oxygen saturation, and lactate. Am J Emerg Med.

[4] Vincent JL (1996). Determination of oxygen delivery and consumption versus cardiac index and oxygen extraction ratio. Crit Care Clin.

[5] Dellinger RP, Levy MM, Rhodes A, Annane D, Gerlach H, Opal SM, Sevransky JE, Sprung CL, Douglas IS, Jaeschke R, Osborn TM, Nunnally ME, Townsend SR, Reinhart K, Kleinpell RM, Angus DC, Deutschman CS, Machado FR, Rubenfeld GD, Webb S, Beale RJ, Vincent JL, Moreno R, Surviving Sepsis Campaign Guidelines Committee including The Pediatric Subgroup (2013). Surviving Sepsis Campaign: international guidelines for management of severe sepsis and septic shock, 2012. Intensive Care Med.

[6] Angus DC, van der Poll T (2013). Severe sepsis and septic shock. N Engl J Med.

[7] Meregalli A, Oliveira RP, Friedman G (2004). Occult hypoperfusion is associated with increased mortality in hemodynamically stable, high-risk, surgical patients. Crit Care.

[8] Rivers E, Nguyen B, Havstad S, Ressler J, Muzzin A, Knoblich B, Peterson E, Tomlanovich M, Early Goal-Directed Therapy Collaborative Group (2001). Early goal-directed therapy in the treatment of severe sepsis and septic shock. N Engl J Med.

[9] Investigators ProCESS, Yealy DM, Kellum JA, Huang DT, Barnato AE, Weissfeld LA, Pike F, Terndrup T, Wang HE, Hou PC, LoVecchio F, Filbin MR, Shapiro NI, Angus DC (2014). A randomized trial of protocol-based care for early septic shock. N Engl J Med.

[10] Peake SL, Delaney A, Bailey M, Bellomo R, Cameron PA, Cooper DJ, Higgins AM, Holdgate A, Howe BD, Webb SA, Williams P, ARISE Investigators, ANZICS Clinical Trials Group (2014). Goal-directed resuscitation for patients with early septic shock. N Engl J Med.

[11] Bloos F, Reinhart K (2005). Venous oximetry. Intensive Care Med.

[12] Friedman G, Backer D, Shahla M, Vincent JL (1998). Oxygen supply dependency can characterize septic shock. Intensive Care Med.

[13] Kandel G, Aberman A (1983). Mixed venous oxygen saturation. Its role in the assessment of the critically ill patient. Arch Intern Med.

[14] Hayes MA, Timmins AC, Yau EH, Palazzo M, Hinds CJ, Watson D (1994). Elevation of systemic oxygen delivery in the treatment of critically ill patients. N Engl J Med.

[15] Gattinoni L, Brazzi L, Pelosi P, Latini R, Tognoni G, Pesenti A (1995). A trial of goal-oriented hemodynamic therapy in critically ill patients. SvO2 Collaborative Group. N Engl J Med.

[16] Alía I, Esteban A, Gordo F, Lorente JA, Diaz C, Rodriguez JA (1999). A randomized and controlled trial of the effect of treatment aimed at maximizing oxygen delivery in patients with severe sepsis or septic shock. Chest.

[17] Pinsky MR, Vincent JL (2005). Let us use the pulmonary artery catheter correctly and only when we need it. Crit Care Med.

[18] Bakker J, Gris P, Coffernils M, Kahn RJ, Vincent JL (1996). Serial blood lactate levels can predict the development of multiple organ failure following septic shock. Am J Surg.

[19] Okorie ON, Dellinger P (2011). Lactate: biomarker and potential therapeutic target. Crit Care Clin.

[20] Nguyen HB, Loomba M, Yang JJ, Jacobsen G, Shah K, Otero RM (2010). Early lactate clearance is associated with biomarkers of inflammation, coagulation, apoptosis, organ dysfunction and mortality in severe sepsis and septic shock. J Inflamm (Lond).

[21] Nguyen HB, Rivers EP, Knoblich BP, Jacobsen G, Muzzin A, Ressler JA (2004). Early lactate clearance is associated with improved outcome in severe sepsis and septic shock. Crit Care Med.

[22] Puskarich MA, Trzeciak S, Shapiro NI, Heffner AC, Kline JA, Jones AE, Emergency Medicine Shock Research Network (EMSHOCKNET) (2011). Outcomes of patients undergoing early sepsis resuscitation for cryptic shock compared with overt shock. Resuscitation.

[23] Jones AE, Shapiro NI, Trzeciak S, Arnold RC, Claremont HA, Kline JA, Emergency Medicine Shock Research Network (EMShockNet) Investigators (2010). Lactate clearance vs central venous oxygen saturation as goals of early sepsis therapy: a randomized clinical trial. JAMA.

[24] Jansen TC, van Bommel J, Schoonderbeek FJ, Sleeswijk Visser SJ, van der Klooster JM, Lima AP, Willemsen SP, Bakker J, LACTATE study group (2010). Early lactate-guided therapy in intensive care unit patients: a multicenter, open-label, randomized controlled trial. Am J Respir Crit Care Med.

[25] Lagoa CE, Figueiredo LF, Cruz RJ, Silva E, Rocha e Silva M (2004). Effects of volume resuscitation on splanchnic perfusion in canine model of severe sepsis induced by live Escherichia coli infusion. Crit Care.

[26] van Haren FM, Sleigh JW, Pickkers P, Van der Hoeven JG (2007). Gastrointestinal perfusion in septic shock. Anaesth Intensive Care.

[27] Gomersall CD, Joynt GM, Freebairn RC, Hung V, Buckley TA, Oh TE (2000). Resuscitation of critically ill patients based on the results of gastric tonometry: a prospective, randomized, controlled trial. Crit Care Med.

[28] Pittman RN (2005). Oxygen transport and exchange in the microcirculation. Microcirculation.

[29] Ince C, Sinaasappel M (1999). Microcirculatory oxygenation and shunting in sepsis and shock. Crit Care Med.

[30] Fink MP (2002). Cytopathic hypoxia. Is oxygen use impaired in sepsis as a result of an acquired intrinsic derangement in cellular respiration?. Crit Care Clin.

[31] Backer D, Orbegozo CD, Donadello K, Vincent JL (2014). Pathophysiology of microcirculatory dysfunction and the pathogenesis of septic shock. Virulence.

[32] Sakr Y, Dubois MJ, Backer D, Creteur J, Vincent JL (2004). Persistent microcirculatory alterations are associated with organ failure and death in patients with septic shock. Crit Care Med.

[33] Trzeciak S, Dellinger RP, Parrillo JE, Guglielmi M, Bajaj J, Abate NL, Arnold RC, Colilla S, Zanotti S, Hollenberg SM, Microcirculatory Alterations in Resuscitation and Shock Investigators (2007). Early microcirculatory perfusion derangements in patients with severe sepsis and septic shock: relationship to hemodynamics, oxygen transport, and survival. Ann Emerg Med.

[34] Trzeciak S, McCoy JV, Phillip Dellinger R, Arnold RC, Rizzuto M, Abate NL, Shapiro NI, Parrillo JE, Hollenberg SM, Microcirculatory Alterations inResuscitation and Shock (MARS) investigators (2008). Early increases in microcirculatory perfusion during protocol-directed resuscitation are associated with reduced multi-organ failure at 24 h in patients with sepsis. Intensive Care Med.

[35] Backer D, Creteur J, Dubois MJ, Sakr Y, Koch M, Verdant C (2006). The effects of dobutamine on microcirculatory alterations in patients with septic shock are independent of its systemic effects. Crit Care Med.

[36] Buwalda M, Ince C (2002). Opening the microcirculation: can vasodilators be useful in sepsis?. Intensive Care Med.

[37] Spronk PE, Ince C, Gardien MJ, Mathura KR, Oudemans-van Straaten HM, Zandstra DF (2002). Nitroglycerin in septic shock after intravascular volume resuscitation. Lancet.

[38] Boerma EC, Koopmans M, Konijn A, Kaiferova K, Bakker AJ, van Roon EN (2010). Effects of nitroglycerin on sublingual microcirculatory blood flow in patients with severe sepsis/septic shock after a strict resuscitation protocol: a double-blind randomized placebo controlled trial. Crit Care Med.

[39] Mathura KR, Vollebregt KC, Boer K, Graaff JC, Ubbink DT, Ince C (2001). Comparison of OPS imaging and conventional capillary microscopy to study the human microcirculation. J Appl Physiol.

