# Supplementary material for: How to choose the therapeutic goals to improve tissue perfusion in septic shock
Source: Einstein (Sao Paulo). 2015 Jul-Sep;13(3):441–7. doi: 10.1590/S1679-45082015RW3148 (PMC4943794; doi:10.1590/S1679-45082015RW3148)
Supplement: Translation

# Translation: Como escolher os alvos terapêuticos para melhorar a perfusão tecidual no choque séptico

**Authors:** Murillo Santucci Cesar de Assuncao, Thiago Domingos Corrêa, Bruno de Arruda Bravim, Eliézer Silva

**Affiliations:** 1 Hospital Israelita Albert Einstein, São Paulo, SP, Brasil.

**Keywords:** Choque séptico, Hemodinâmica, Ressuscitação, Sepse, Vasoconstritores, Microcirculação

## Abstract

O reconhecimento e o tratamento precoce da sepse grave e do choque séptico é a chave para o sucesso terapêutico. Quanto maior o atraso no início do tratamento, pior é o prognóstico, em decorrência da hipoperfusão tecidual persistente, e do consequente desenvolvimento e agravamento das disfunções orgânicas. Um dos principais mecanismos responsáveis pelo desenvolvimento da disfunção celular é a hipóxia. A adequação do fluxo sanguíneo tecidual e, consequentemente, da oferta de oxigênio à demanda metabólica, de acordo com a avaliação do índice cardíaco e da taxa de extração de oxigênio, deve ser realizada durante a ressuscitação, principalmente nos pacientes de alta complexidade. Novas tecnologias, de fácil manuseio à beira do leito, e novos estudos, que avaliem diretamente o impacto da otimização dos parâmetros macro-hemodinâmicos na microcirculação e no desfecho clínico dos pacientes sépticos, são necessários.

## INTRODUÇÃO

A sepse caracteriza-se pelo padrão heterogêneo de fluxo sanguíneo na microcirculação, pela hipoperfusão tecidual e pela incapacidade das células extraírem e utilizarem adequadamente o oxigênio, o que compromete o metabolismo aeróbio celular.^(1-3)^ O desequilíbrio entre a oferta de oxigênio (DO_2_) e a demanda metabólica celular é o que caracteriza o estado de choque.^(4)^

A sepse, associada a sinais de disfunção de um ou mais órgãos, recebe a denominação de “sepse grave”. O choque séptico é definido pela necessidade de vasopressor para manutenção da pressão de perfusão após reposição volêmica adequada.^(5)^

A sepse compromete o sistema cardiovascular por diversos mecanismos, que frequentemente resultam em alterações da perfusão tecidual. A hipoperfusão tecidual contribui para o desenvolvimento de disfunção celular, a qual pode progredir para disfunção de múltiplos órgãos e óbito.^(6)^ O objetivo principal da terapêutica nos estados de choque é o restabelecimento da DO_2 _aos tecidos, para adequar a demanda de oxigênio tecidual. Assim, intervenções rápidas e precoces, com o objetivo de reverter a hipoperfusão tecidual, têm sido associadas à redução da mortalidade em pacientes sépticos.^(5)^

O objetivo desta revisão da literatura é discutir os principais parâmetros hemodinâmicos e laboratoriais que norteiam a abordagem do paciente com choque séptico à beira do leito.

## PARÂMETROS CLÍNICOS E MACRO-HEMODINÂMICOS

É sabido que os parâmetros clínicos e macro-hemodinâmicos apresentam baixa correlação com o estado da perfusão tecidual. Em 1996, Rady et al. demonstraram a presença de hipoperfusão tecidual oculta pela presença de hiperlactatemia ou redução da saturação venosa central de oxigênio (SvcO_2_).^(3)^ Pacientes graves foram submetidos à ressuscitação inicial com fluidos, seguida da estabilização dos parâmetros clínicos. Aproximadamente 86% dos pacientes avaliados apresentavam hiperlactatemia ou SvcO_2_ <65% após a estabilização do parâmetros clínicos.^(3)^ Isso foi caracterizado como hipoperfusão tecidual oculta ou choque crítico.^(7)^

Tais achados evidenciam que os parâmetros clínicos não devem ser utilizados isoladamente como meta terapêutica na ressuscitação de pacientes graves. O estudo de Rady et al.^(3)^ precedeu o *Early Goal-Directed Therapy*, o qual demonstrou que um grupo de intervenções realizadas precocemente envolvendo infusão de fluidos, uso de vasopressores, inotrópicos e de transfusão de hemácias, tendo como meta a SvcO_2_≥70%, reduziu a mortalidade em pacientes com sepse grave e choque séptico.^(8)^

Os resultados deste estudo são considerados a maior evidência disponível de que a otimização da perfusão tecidual deve ser realizada precocemente e adicionada como uma meta no tratamento dessa população de pacientes graves.^(8) ^Todavia, tal benefício não foi observado em dois grandes estudos publicados recentemente.^(9,10)^ Os resultados negativos obtidos nestes dois estudos podem ser, ao menos parcialmente, justificados pelo fato de os pacientes incluídos terem recebido infusão de fluidos precocemente, antes da randomização.^(9,10)^ O estudo de Rivers et al. foi o grande divisor de águas na abordagem de pacientes graves, sendo sua principal mensagem a importância da abordagem precoce dos pacientes graves.^(8)^ Após este estudo e com a disseminação da informação sobre a necessidade do reconhecimento e tratamento precoces dos pacientes com sepse grave ou choque séptico, torna-se difícil comparar uma conduta que mudou a abordagem terapêutica. Além disso, considera-se, na atualidade, antiético o atraso no reconhecimento e tratamento desta população de pacientes graves.

Tanto o estudo ProCESS como o ARISE (*Australasian Resuscitation in Sepsis Evaluation*) tiveram como critério de inclusão a necessidade de infusão de fluidos e início da administração de antibióticos de amplo espectro.^(9,10) ^Essas medidas, que enfatizam a importância da precocidade do tratamento, certamente contribuíram para a observação de mortalidade semelhante entre diversos grupos estudados.^(9,10)^ A precocidade em iniciar o tratamento é o que difere estes dois estudos do estudo de Rivers et al.^(8-10)^

Diversos parâmetros e marcadores de fluxo sanguíneo e de perfusão tecidual sistêmica estão disponíveis à beira do leito, como a saturação venosa mista de oxigênio (SvO_2_), o clareamento de lactato, a DO_2_ e o consumo de oxigênio (VO_2_). A interpretação desses marcadores se faz necessária para a adequação da ressuscitação e o restabelecimento da perfusão tecidual.

## PARÂMETROS DE OXIGENAÇÃO

A SvO_2_ reflete o total de sangue oxigenado que retorna para o coração direito pela drenagem de sangue no átrio direito, pela veia cava superior, veia cava inferior, seio coronário e rede de Tebesius.^(11)^ A SvO_2 _é mensurada em amostra de sangue coletada da artéria pulmonar. O sangue venoso oriundo de várias partes do organismo começa a se homogeneizar no átrio direito e, à medida que progride em direção aos pulmões, torna-se cada vez mais homogêneo. Ao chegar na artéria pulmonar, o sangue venoso de todas as partes do organismo encontra-se totalmente “misturado”, ou seja, homogeneizado por completo, recebendo, assim, a denominação de “sangue venoso misto”.^(11)^ A SvcO_2_, por sua vez, corresponde à saturação de oxigênio pela hemoglobina do sangue, que se encontra na desembocadura da veia cava superior no átrio. A SvcO_2 _reflete a quantidade de oxigênio que retorna ao coração direito oriunda dos membros superiores, do pescoço e da cabeça.^(11)^

Em indivíduos sadios, a SvcO_2_ apresenta valor médio de 77%, com variação entre 66 a 84%, enquanto a SvO_2_ apresenta valor médio de 78%, com variação entre 73 a 85%. Assim, em situações de normalidade, a SvcO_2_ apresenta valores inferiores em relação aos da SvO_2_.^(11)^ No entanto, devido ao maior VO_2 _na circulação esplâncnica, órgãos intra-abdominais e membros inferiores, nos estados de choque ocorre uma inversão dessa tendência, e a SvcO_2_ passa a apresentar valores superiores aos da SvO_2_.^(11)^

Assumindo que a saturação arterial de oxigênio encontra-se constante, a SvO_2_ apresenta relação direta com o débito cardíaco (Figura 1). Além do débito cardíaco (fluxo sistêmico), a SvO_2_ apresenta relação inversa com a taxa de extração de oxigênio (TEO_2_) (Figura 1). A TEO_2_, por sua vez, representa a quantidade de oxigênio que as células conseguem extrair a partir de uma determinada quantidade de oxigênio que lhes é ofertada e representa a relação entre o VO_2_ e a DO_2_.^(4)^

**Figura 1:** Relações entre oferta, consumo e taxa de extração de oxigênio, saturação venosa mista de oxigênio, lactato arterial e gradiente de pressão parcial de gás carbônico da mucosa gástrica

A DO_2_ está relacionada ao fluxo sanguíneo e à quantidade de oxigênio que se encontra ligada à hemoglobina e dissolvida no plasma, sendo definida como a quantidade de oxigênio que chega às células para a atender a demanda metabólica do organismo. A quantidade de oxigênio que as células consomem se relaciona com a capacidade de extração celular de oxigênio. Dessa forma, o VO_2_ é definido pela quantidade de oxigênio utilizada pelas células para a produção de energia para suprir a demanda metabólica.^(4)^

Fisiologicamente, todas as vezes em que houver redução da DO_2_, haverá um aumento da extração tecidual de oxigênio para manter o VO_2_ estável (Figura 1). Nessa situação, é esperado que a SvO_2_ diminua, visto que ela representa o total de sangue oxigenado que retorna da circulação sistêmica para o coração direito e reflete o balanço entre o VO_2_ e a DO_2_. Porém quando reduções da DO_2_ forem acompanhadas paralelamente pela diminuição do VO_2_, inicia-se mecanismo de anaerobiose para atender à demanda metabólica do organismo. Nesse cenário, o aumento da extração de oxigênio não é capaz de suprir e nem de manter as necessidades metabólicas dependentes de oxigênio. Quando esse processo se inicia, a DO_2_ é chamada de DO_2_ crítica e se estabelece a dependência VO_2_/DO_2_ (Figura 1).^(4)^

Apesar da dependência VO_2_/DO_2_ não estar frequentemente associada a estados de alto metabolismo como na sepse, ela pode ainda estar presente ao final da ressuscitação guiada pela ScvO_2_.^(4,12)^ A otimização do fluxo sanguíneo sistêmico guiado pela SvcO_2_ durante as seis horas iniciais de ressuscitação de pacientes com sepse grave ou choque séptico reduziu a mortalidade dessa população de pacientes quando comparada ao grupo controle.^(8)^ Todavia, a SvcO_2 _representa um marcador sistêmico de adequação do fluxo sanguíneo, e valores de SvcO_2_≥70% não significam que o fluxo sanguíneo regional, em cada órgão e tecido, está otimizado por completo.^(8)^ Para ter a certeza de que a ressuscitação inicial atingiu seu objetivo, deve-se descartar a presença de défice de oxigênio, o qual é caracterizado pelo aumento do VO_2_ em consequência da otimização ou do aumento do fluxo sanguíneo.^(4)^

O que deve ser sempre questionado durante a ressuscitação é se o fluxo sanguíneo obtido é compatível com a demanda metabólica de oxigênio. Por isso, tem-se sido sugerido como método para avaliar a existência de défice de oxigênio um teste, em que se aumentam o débito cardíaco e, consequentemente, fluxo de oxigênio tecidual. A ausência de alteração da SvO_2_ após aumento do débito cardíaco sugere aumento do VO_2,_ significando que existe ainda um défice de oxigênio e, portanto, que a ressuscitação ainda não atingiu o objetivo principal.^(13-17)^

Atingir o ajuste da relação DO_2_/VO é o racional para a correção da hipoperfusão. A avaliação da relação entre a TEO_2_ e o índice cardíaco permite otimizar o VO_2_.^(4)^ Objetivando adequar a DO_2_ ao VO_2_, podem ser necessários valores elevados de DO_2_. Essa alta DO_2 _para um alto VO_2_ não é sinônimo de terapia supranormal. Na terapia supranormal, valores elevados de DO_2_ preestabelecidos são objetivados, independentemente da relação DO_2_/VO_2._^(14-16)^

Na fase aguda do choque séptico, a dependência DO_2_/VO_2_ pode ser identificada e corrigida.^(12)^ A adequação da relação DO_2_/VO_2 _é, desta forma, o *endpoint *mais importante da ressuscitação. Ao se realizarem incrementos do débito cardíaco, que proporcionam aumento do VO_2_, pode-se inferir que áreas que não estavam sendo perfundidas de forma adequada passaram a ser e que ocorreu um aumento do VO_2_, isto é, um recrutamento da microcirculação (Figura 2). A adequação do índice cardíaco à demanda metabólica, associada à normalização dos parâmetros de perfusão sistêmicos, como a SvO_2_ ou o lactato, permitem concluir que a ressuscitação foi concluída com êxito.

**Figura 2:** Comportamento da saturação venosa mista de oxigênio, de acordo com a oferta e o consumo de oxigênio

Baseando-se nos três pilares que apresentam maior impacto no débito cardíaco, ou seja, a pré-carga, a contratilidade e a pós-carga, pode-se iniciar a otimização de fluxo sanguíneo pela avaliação da responsividade à infusão de fluidos. O benefício da infusão de fluidos ocorre quando o paciente encontra-se na parte ascendente da curva de Frank-Starling, isto é, o paciente ainda apresenta pré-carga recrutável. Caso o paciente se encontre no platô da curva de Frank-Starling, ou seja, não responder a infusão de fluidos, é possível utilizar agentes inotrópicos, como a dobutamina, com dose inicial de 2,5mcg/kg/min, e avaliar o ganho de fluxo pelo aumento do índice cardíaco, sempre associado a análise da TEO_2_, para inferir no comportamento do VO_2_. Assim, independente da estratégia adotada, o aumento do fluxo sanguíneo com a manutenção dos valores de SvO_2_, traduzidos pelo aumento da TEO_2_, implicaria no aumento do VO_2_ (Figuras 1 e 2).

A análise integrada de vários parâmetros hemodinâmicos parece ser a mais adequada e útil no tratamento de pacientes com choque, em comparação a utilização isolada de apenas uma das variáveis.

## *CLEARANCE *DE LACTATO

O lactato é um marcador de perfusão tecidual sistêmico e encontra-se elevado nos casos de hipoperfusão tecidual. A demora na normalização do lactato em pacientes graves é um marcador de prognóstico desfavorável. Nesse contexto, a duração da hiperlactatemia é mais importante que o valor inicial do lactato.^(18)^ Todavia, na sepse, existem outros fatores, além da hipoperfusão sistêmica, que podem contribuir com a hiperlactatemia, como a disfunção da enzima piruvato desidrogenase, a disfunção mitocondrial e a presença de disfunção hepática e renal.^(19)^

O clareamento ou *clearance* de lactato é representado pela porcentagem da redução dos níveis de lactato após o início da ressuscitação. O clareamento de lactato ≥10% foi associado a melhor prognóstico em pacientes graves, com redução da mortalidade entre pacientes com sepse grave e choque séptico.^(20)^

O *clearence* de lactato representa um parâmetro a ser monitorado durante a otimização da perfusão tecidual sistêmica.^(20-22)^ As diretrizes da *Surviving Sepsis Campaign *recomendam o acompanhamento do *clearance* de lactato e sua normalização o mais precoce possível, durante a fase de ressuscitação inicial (Figura 3).^(5)^

**Figura 3:** Algoritmo de ressuscitação protocolado guiado por metas para pacientes com choque séptico

O *clearance* de lactato pode ser utilizado como substituto da monitoração da SvcO_2_, para otimização do fluxo sanguíneo sistêmico durante a fase inicial de ressuscitação de pacientes com sepse grave e choque séptico.^(23)^ O emprego de um protocolo agressivo de ressuscitação, com o objetivo de clarear o lactato em pelo menos 20%, a cada 2 horas, reduziu significativamente o tempo de internação no hospital, na unidade de terapia intensiva (UTI) e a mortalidade hospitalar.^(24)^ No entanto, ainda não existem evidências de que a normalização do lactato apresente benefícios adicionais quando comparada com a ressuscitação guiada apenas pela pressão venosa central (PVC), pressão arterial média (PAM), diurese e SvcO_2_.

Todavia, a normalização dos níveis de lactato não pode ser definida como *endpoint* de ressuscitação da microcirculação. O lactato medido corresponde a diluição da produção global de lactato por todos os órgãos. Isso significa que pode existir hipoperfusão tecidual regional, ainda que os níveis plasmáticos de lactato estejam normalizados.

## PARÂMETROS MICRO-HEMODINÂMICOS

### Fluxo sanguíneo regional

Na sepse, as variáveis de perfusão regional são alteradas precocemente em relação às variáveis sistêmicas e, após a ressuscitação, as variáveis regionais podem ser as últimas a se normalizarem.^(25,26)^ Estas variáveis são o pH da mucosa gástrica e o gradiente entre a pressão parcial de gás carbônico da mucosa gástrica (Gap CO_2_) e a pressão arterial parcial de gás carbônico (PCO_2_ gap).

A tonometria gástrica ou a capnometria sublingual podem ser utilizadas para detectar precocemente a hipoperfusão tecidual. O aumento do CO_2_ gap pode refletir fluxo sanguíneo anormal na mucosa gástrica. Dessa forma, a monitoração pela tonometria gástrica poderia ajudar na identificação e na correção das anormalidades circulatórias regionais.

Entretanto, nenhum estudo conseguiu confirmar o benefício do emprego dessas técnicas como guia terapêutico, principalmente em pacientes sépticos, sendo que, atualmente, a tonometria gástrica é uma técnica utilizada apenas em modelos experimentais de choque.^(27)^

### Disfunção da microcirculação: um novo campo a ser explorado

Em condições normais, somente 30% dos capilares são perfundidos. Dessa forma é possível imaginar que, mesmo em condições normais, a microcirculação não apresenta oxigenação homogênea. Para conseguir uma oxigenação homogênea, os tecidos necessitam recrutar capilares, o que normalmente ocorre em condições de estresse metabólico.^(28)^

A disfunção da microcirculação na sepse grave e no choque séptico é caracterizada pela presença de anormalidades no fluxo sanguíneo, podendo coexistir áreas com fluxo sanguíneo elevado e perfusão tecidual normal e áreas com perfusão alterada. A presença de capilares hipoperfundidos pode comprometer a oxigenação tecidual e contribuir para o desenvolvimento da disfunção e da falência de múltiplos órgãos. Postula-se atualmente que a reversão destes distúrbios microcirculatórios possa ser um alvo terapêutico a ser almejado durante a ressuscitação da sepse, o que propiciaria a reversão das áreas de hipóxia decorrente da hipoperfusão tecidual desencadeada pelo grande número de *shunts*. Assim, a disfunção da microcirculação passou a ser uma preocupação no tratamento de pacientes sépticos e sua reversão tem se tornado um alvo terapêutico na ressuscitação do choque séptico.

Foram levantadas ainda duas hipóteses sobre a origem da disfunção da microcirculação no choque séptico. Primeiro, a alteração e a perda da autorregulação da microcirculação resultaria em perfusão heterogênea, o que promoveria áreas de hypoxia, resultando em aumento das áreas de *shunt*.^(29)^ Segundo, a disfunção mitocondrial pode ser induzida pela sepse, que poderia alterar a capacidade mitocondrial de utilizar o oxigênio a despeito da adequada oxigenação tecidual (hipóxia citopática).^(30)^ Todavia, a extensa lesão endotelial, a liberação de mediadores inflamatórios, a ativação da coagulação e as alterações no metabolismo celular do oxigênio podem contribuir para o desenvolvimento da disfunção da microcirculação em pacientes com choque séptico.^(31)^

O grau de disfunção da microcirculação é um preditor independente de sobrevida. Pacientes sépticos com graves alterações na microcirculação sublingual apresentam pior prognóstico em relação àqueles que não apresentam alterações significativas da microcirculação.^(1,32)^ Em estudo observacional realizado durante as primeiras seis horas de ressuscitação de pacientes com sepse grave e choque séptico, o comprometimento dos índices de perfusão da microcirculação foram associados com redução da sobrevida.^(33)^ Neste estudo, a melhora do fluxo sanguíneo na microcirculação foi associada a menor incidência de disfunção orgânica em 24 horas, sem que alterações significantes nos parâmetros hemodinâmicos sistêmicos fossem detectadas.^(34)^ Desse modo, o recrutamento da microcirculação, com o objetivo de melhorar a oxigenação cellular, possui o potencial de minimizar a progressão da disfunção orgânica, ao satisfazer e adequar a oxigenação celular de acordo com a demanda metabólica.

Algumas drogas vasoativas podem otimizar a perfusão da microcirculação. A dobutamina melhora a perfusão capilar, independentemente das alterações dos parâmetros macro-hemodinâmicos. A redução dos níveis de lactato se correlaciona com a melhora da microcirculação, independentemente do índice cardíaco.^(35)^ Entretanto o lactato não é um marcador de ressuscitação da microcirculação.

Uma outra maneira de promover o recrutamento de capilares é a utilização de agentes vasodilatadores. Na sepse, foi proposto que o emprego de vasodilatadores pode aumentar o fluxo sanguíneo na microcirculação, aumentando a perfusão em áreas de hipóxia.^(36)^ No entanto, ainda existem controvérsias sobre a utilização de vasodilatadores para recrutamento da microcirculação. Os vasodilatadores (nitroglicerina e nitroprussiato de sódio) são agentes doadores de óxido nítrico, o que poderia contribuir com a disfunção celular da sepse. Enquanto Spronk et al. demonstraram melhora da perfusão da microcirculação sublingual com infusão de nitroglicerina,^(37)^ Boerma et al. reportaram que o uso de nitroglicerina não melhorou a perfusão da microcirculação sublingual em comparação a administração de placebo.^(38)^ Todavia, houve tendência ao aumento da mortalidade hospitalar no grupo de pacientes que recebeu nitroglicerina. Entretanto, este estudo não teve poder suficiente para confirmar a associação entra a administração de nitroglicerina e aumento da mortalidade. Outro achado interessante deste estudo foi que os pacientes que receberam nitroglicerina apresentaram menor número de disfunções orgânicas quando comparados com o grupo placebo.^(38)^

A ressuscitação e o recrutamento da microcirculação com administração de fluídos, dobutamina e vasodilatadores pode ser uma estratégia promissora. No entanto, a avaliação da microcirculação à beira leito continua limitada a protocolos de pesquisa, apesar de, nos últimos anos, novos equipamentos de avaliação da microcirculação terem sido desenvolvidos, facilitando o emprego dessa tecnologia a beira do leito.^(39)^ Além do aprimoramento das técnicas disponíveis, ainda há necessidade de ensaios clínicos adequados para comprovar o real benefício dessa estratégia de ressuscitação.

## CONCLUSÃO

A otimização precoce da oxigenação tecidual é mandatória na ressuscitação dos pacientes com sepse grave e choque séptico. A utilização de um protocolo de ressuscitação precoce guiado por metas, que inclua a saturação venosa central de oxigênio ou o *clearance* de lactato como parâmetros de adequação do fluxo sanguíneo e, consequentemente, da oferta de oxigênio a demanda metabólica, é altamente recomendada pelas diretrizes que norteiam a ressuscitação de pacientes com sepse grave e choque séptico.

Novas tecnologias para visualização direta da microcirculação, de fácil manuseio à beira do leito e custo-efetivas são ainda necessárias para maior utilização na prática clínica diária. Além disso, novos estudos, com objetivo de avaliar o impacto da otimização dos parâmetros macro-hemodinâmicos na microcirculação e no desfecho dos pacientes sépticos, ainda são necessários.
